# Oral Health Literacy, Attitude, and Practices and Their Influence on Oral Health Status Among Sugar Mill Workers in Villupuram District: A Cross-Sectional Study

**DOI:** 10.7759/cureus.94760

**Published:** 2025-10-17

**Authors:** Sindhumathi Kannan, Jagannatha G Venkatarayappa, Cyril H Benedict, Nagaland Tirupati, Vidhya G, Kokila S K

**Affiliations:** 1 Public Health Dentistry, Chettinad Dental College and Research Institute, Kelambakkam, IND

**Keywords:** community periodontal index, oral health, oral health literacy, positive attitude, practices, dental caries

## Abstract

Background: Oral diseases constitute a significant component of the global disease burden; however, they remain largely preventable through appropriate knowledge acquisition and the adoption of positive health behaviors. This study aimed to evaluate oral health literacy (OHL), attitude, and practices and examine their association with oral health status among sugar mill workers.

Methodology: This cross-sectional study was conducted among 260 participants aged 18-60 years in Villupuram District. A validated questionnaire (Health Literacy in Dentistry Scale (HeLD-14)) about OHL, attitude, and practices was used to collect data, followed by which oral health status was assessed using the Decayed, Missing, and Filled Teeth (DMFT) index and the Community Periodontal Index/Loss of Attachment (CPI/LOA) indices. Descriptive and inferential statistical analyses were performed using the Mann-Whitney U test, chi-square test, and Fisher’s exact test in IBM SPSS Statistics for Windows (IBM Corp., Armonk, NY, USA).

Results: The mean age of the participants was 37.5 ± 7.5 years, with 75.8% of them being males. Participants exhibiting higher OHL demonstrated significantly lower DMFT scores and better periodontal status (p < 0.05). Similarly, positive oral health attitude and practices were significantly associated with better CPI scores (p < 0.05). However, no significant association was found between attitude, practices, and LOA or DMFT scores (p > 0.05).

Conclusion: This study concludes that higher OHL was significantly associated with lower caries experience and better periodontal status among the participants. Similarly, attitude and practices showed a positive influence on periodontal health. Enhancing OHL may therefore be a key strategy for improving oral health in this population.

## Introduction

Oral health is an integral component of general health and exerts a significant influence on overall quality of life (QoL). According to the WHO Global Oral Health Status Report (2022), oral diseases affect about half of the global population, with the highest burden in low- and middle-income countries, impacting approximately 3.5 billion people [1]. Optimal oral health is largely dependent on an individual’s ability to comprehend and implement health information delivered through both verbal and written communications [2]. Literacy, as a dynamic and evolving construct, forms a core element of communication and thereby plays a significant role in ensuring effective healthcare services [3].

Oral health literacy (OHL), a subset of health literacy, is defined as the degree to which individuals have the capacity to obtain, process, and understand basic oral health information and services needed to make appropriate oral health decisions, thus playing a pivotal role in informed decision-making regarding oral hygiene practices and utilization of dental care services [4,5]. Moreover, it serves as a significant predictor that healthcare providers should consider in clinical practice [6]. A study by Tyagi et al. reported that limited literacy has been associated with unhealthy behaviors, reduced utilization of preventive services, increased rates of hospitalization and chronic diseases, higher healthcare expenditures, and overall poor health outcomes compared to individuals with higher literacy levels [7].

Enhancing OHL is therefore considered a priority for improving oral health outcomes, especially in socially disadvantaged groups [8]. Although oral diseases are largely preventable and manageable, the effectiveness of treatment varies considerably among individuals, primarily due to differences in awareness and adherence to oral health practices. Ying et al. reported that an individual's OHL directly influences their oral health behaviors, with high OHL promoting healthy oral hygiene practices, and lower literacy fosters poor hygiene practices and a preference for curative rather than preventive interventions [9].

Pavithra et al. reported that although the majority of individuals used a toothbrush and toothpaste, there was limited knowledge about proper brushing techniques, optimal brushing duration, appropriate brush replacement frequency, and the use of additional oral hygiene aids [10]. Notwithstanding adequate levels of individual literacy, favorable attitudes, and adherence to healthy practices, the occupational environment exerts a profound and independent influence on both general and oral health. This highlights that workplace-related exposures, physical conditions, and psychosocial factors may significantly shape health outcomes, often overriding or modifying the benefits conferred by personal determinants. Studies have highlighted that various components of the stomatognathic system are particularly susceptible to occupational hazards, which may adversely affect the teeth, jawbones, periodontal tissues, tongue, lips, and oral mucosa [11].

One such occupationally vulnerable sector is the sugar industry [12]. India, being the world's second-largest sugar producer, accounts for around 15% of global sugar and 25% of global sugarcane production [13]. Previous studies reported that individuals working in confectioneries, bakeries, sugar refineries, and sugar mills exhibit a significantly higher prevalence of dental caries due to prolonged exposure to elevated concentrations of sugar dust in their occupational environment [14-16].

Although limited, OHL contributes significantly to oral health disparities [17], and its association with actual oral health status in high-risk occupational groups, such as sugar mill workers in India, remains underexplored [12]. Therefore, the objectives of the present study are to assess the OHL, attitude, practices, and oral health status of sugar mill workers in the Villupuram District of Tamil Nadu and thereby to explore the association between them.

## Materials and methods

The cross-sectional study was conducted among 260 sugar mill workers in Villupuram District. Ethical clearance was obtained from the Institutional Human Ethics Committee (IHEC) of Chettinad Dental College and Research Institute (IHEC-CDCRI/2024/STU-0096), and permissions were obtained from the General Managers of respective sugar mills before the start of the study. Sample size was calculated using G*Power software (version 3.1.9.7, Heinrich-Heine-Universität Düsseldorf, Düsseldorf, Germany) based on the findings from a previous study [17], with a level of significance (α) at 5% and power (1-β) of the study at 95%. The estimated sample size was 245 participants. Anticipating a non-response rate of 5%, 257 samples were considered the final sample size, rounded off to 260.

A simple random sampling technique was employed to select the participants. Of the 46 sugar industries in Tamil Nadu, the Villupuram District accounted for the largest proportion. Therefore, a comprehensive list of these sugar mills was prepared, and two mills were randomly selected through the lottery method. Eligible participants from the selected mills were recruited after obtaining written informed consent. Employees aged 18-60 years who were present on the day of examination were included, while those with less than one year of employment in the sugar mill or with systemic diseases, psychological disorders, or physical disabilities were excluded.

**Figure 1:**
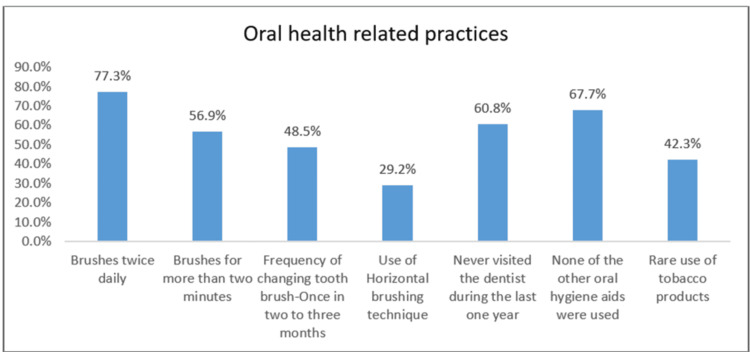
Distribution of sociodemographic characteristics among the study participants

OHL was assessed using a pre-validated questionnaire, the Health Literacy in Dentistry (HeLD)-14 scale [18], which comprises 14 questions under seven domains, such as access, understanding, support, utilization, economic barrier, receptivity, and communication, to yield a total score ranging from 0 to 56, with higher scores indicating higher OHL (Supplemental material 1). Oral health-related attitude was measured using a five-item, self-developed and validated questionnaire addressing individuals' beliefs, perceptions, and behaviors toward maintaining oral hygiene and seeking dental care (Supplemental material 2). Oral health-related practices were assessed using a seven-item, self-developed and validated questionnaire relating to preventive care practices, appropriate brushing and flossing techniques, and routine dental visits (Figure 1). The content validation of the questionnaire was done using Lawshe's Content Validity Ratio (CVR) methods [19] prior to data collection to ensure clarity, comprehensiveness, and relevance. Of 12 items, all the questions had a CVR above the required threshold, and hence, all the questions were retained in the final tool for conducting the study.

Data were collected through face-to-face interviews at the respective sugar mills, and ADA Type III dental examination was employed to assess the oral health status using the Decayed, Missing, and Filled teeth (DMFT) index [20] and Community Periodontal index/ Loss of Attachment (CPI/LOA) indices. Prior to data collection, the principal investigator underwent training and calibration in assessing the oral health status to ensure accuracy, reliability, and reproducibility of the findings of the study.

The collected data were coded and entered into Microsoft Office Excel (Microsoft Corp., Redmond, WA, USA), and statistical analysis was carried out using the IBM SPSS Statistics for Windows, Version 21.0 (Released 2012; IBM Corp., Armonk, NY, USA). Descriptive statistics were used to summarize the data, while inferential statistics, including the Mann-Whitney U test, chi-square test, and Fisher’s exact test, were employed to examine the associations between OHL, attitude, and practices and their influence on oral health status. A p-value < 0.05 was considered statistically significant.

## Results

Of the 260 participants, 75.8% were male and 24.2% were female. The mean age of the study participants was 37.55 ± 7.55 years. Socioeconomic status (SES) of the participants was assessed using the Modified Kuppuswamy Socioeconomic Status Scale for Industrial workers (income scores adjusted according to January 2025) [21], where nearly half of the participants (42.3%) belonged to the upper lower socioeconomic class, and most of them (85.8%) were married (Table 1).

**Table 1:** Distribution of sociodemographic characteristics among the study participants

Variables	Stratification	n (%)
Age (in years)	20-29	49 (18.8)
	30-39	112 (43.1)
	40-49	88 (33.8)
	50-59	11 (4.2)
Gender	Male	197 (75.8)
	Female	63 (24.2)
Socioeconomic status	I (upper)	11 (4.2)
	II (upper middle)	18 (6.9)
	III (lower middle)	96 (36.9)
	IV (upper lower)	110 (42.3)
	V (lower)	25 (9.6)

The mean OHL score was found to be 30.96 ± 14.17. Median split (median = 31) was used to categorize the OHL into low and high, where nearly an equal proportion of the participants had low (50.8%) and high (49.2%) OHL levels. With respect to oral health-related attitude and practices, more than half of the participants (68.5%) had an inadequate attitude, and only 31.5% had an adequate attitude toward oral health. Around 56.2% of the participants had inadequate and 43.8% had adequate oral health practices (Table 2).

**Table 2:** Distribution of OHL, oral health-related attitude, and practices among the study participants

Variables	Stratification	n (%)
Oral health literacy (OHL)	Low oral health literacy	132 (50.8)
	High oral health literacy	128 (49.2)
Oral health-related attitude	Inadequate	178 (68.5)
	Adequate	82 (31.5)
Oral health practices	Inadequate	146 (56.2)
	Adequate	114 (43.8)

The oral health profile of sugar mill workers revealed that there was a statistically significant difference in the mean DMFT scores between the participants with low and high OHL, in which those with high OHL had significantly lower mean DMFT (p = 0.001) scores. Similarly, subjects with high OHL had a healthy periodontal status (p = 0.001) and significantly less attachment loss (p = 0.004) when compared to those with low OHL (Table 3 and Table 4).

**Table 3:** Association between OHL, attitude, practices, and dental caries status (DMFT) among the study participants Mann-Whitney U test was used. *p < 0.05 is considered statistically significant. DMFT: Decayed, Missing, and Filled Teeth.

Variable	Stratification	DMFT		Mean rank	Sum of rank	Z value	p-value
		Mean	SD				
Oral health literacy (OHL)	Low OHL	3.07	1.92	147.11	19418.50	-3.682	0.001*
	High OHL	2.22	1.43	113.37	14511.50		
Oral health attitude	Inadequate	2.79	1.87	135.42	24104.00	-1.581	0.114
	Adequate	2.35	1.42	119.83	9826.00		
Oral health practices	Inadequate	2.63	1.70	131.33	19174.00	-0.205	0.838
	Adequate	2.68	1.82	129.44	14756.00		

**Table 4:** Association between OHL and Community Periodontal Index (CPI) and Loss of Attachment (LOA) scores among the study participants Chi-square and Fisher’s exact tests were used. *p < 0.05 was considered statistically significant.

Variable		Community Periodontal Index (CPI) scores												
		Healthy	Bleeding		Calculus		Pocket 4-5 mm		Pocket 6 mm or more	ꭓ2 value		Cramer’s V		p-value
OHL	Low OHL	8 (6.1%)	34 (25.8%)		58 (43.9%)		20 (15.2%)		12 (9.1%)	31.850		0.350		0.001*
	High OHL	42 (32.8%)	30 (23.4%)		38 (29.7%)		13 (10.2%)		5 (3.9%)					
Variable		Loss of Attachment (LOA) scores												
		0-3 mm		4-5 mm		6-8 mm		9-11 mm		ꭓ2 value	Cramer’s V		p-value	
OHL	Low OHL	50 (37.9%)		65 (49.2%)		15 (11.4%)		2 (1.5%)		12.284	0.217		0.004*	
	High OHL	74 (57.8%)		45 (35.2%)		6 (4.7%)		3 (2.3%)						

A significant association was observed between both oral health-related attitudes and practices and CPI scores. Participants with adequate attitudes (35.4%) and practices (36.8%) demonstrated a higher proportion of healthy periodontal status compared to those with inadequate attitudes (11.8%) and practices (5.5%) (attitude: χ² = 22.285, p = 0.001; practices: χ² = 42.849, p = 0.001). However, no significant association was found between either attitude or practices and the LOA component of the CPI-LOA index (attitude: χ² = 1.290, p = 0.740; practices: χ² = 4.726, p = 0.188), suggesting that while current periodontal condition is influenced by these factors, cumulative attachment loss is not (Table 5 and Table 6).

**Table 5:** Association between oral health-related attitude and Community Periodontal Index (CPI) and Loss of Attachment (LOA) scores among the study participants Chi-square and Fisher’s exact tests were used. *p < 0.05 is considered statistically significant.

Variable		Community Periodontal Index (CPI) scores									
		Healthy	Bleeding	Calculus		Pocket 4-5 mm		Pocket 6 mm or more	ꭓ2 value	Cramer’s V	p-value
Attitude	Inadequate	21 (11.8%)	44 (24.7%)	74 (41.6%)		27 (15.2%)		12 (6.7%)	22.285	0.293	0.001*
	Adequate	29 (35.4%)	20 (24.4%)	22 (26.8%)		6 (7.3%)		5 (6.1%)			
Variable		Loss of Attachment (LOA) scores									
		0-3 mm	4-5 mm		6-8 mm		9-11 mm		ꭓ2 value	Cramer’s V	p-value
Attitude	Inadequate	82 (46.1%)	77 (43.3%)		16 (9.0%)		3 (1.7%)		1.290	0.067	0.740
	Adequate	42 (51.2%)	33 (40.2%)		5 (6.1%)		2 (2.4%)				

**Table 6:** Association between oral health practices and Community Periodontal Index (CPI) and Loss of Attachment (LOA) scores among the study participants Chi-square and Fisher’s exact tests were used. *p < 0.05 is considered statistically significant.

Variable		Community Periodontal Index (CPI) scores									
		Healthy	Bleeding	Calculus		Pocket 4-5 mm		Pocket 6 mm or more	ꭓ2 value	Cramer’s V	p-value
Practices	Inadequate	8 (5.5%)	45 (30.8%)	58 (39.7%)		24 (16.4%)		11 (7.5%)	42.849	0.406	0.001*
	Adequate	42 (36.8%)	19 (16.7%)	38 (33.3%)		9 (7.9%)		6 (5.3%)			
Variable		Loss of Attachment (LOA) scores									
		0-3 mm	4-5 mm		6-8 mm		9-11 mm		ꭓ2 value	Cramer’s V	p-value
Practices	Inadequate	61 (41.8%)	69 (47.3%)		13 (8.9%)		3 (2.1%)		4.726	0.134	0.188
	Adequate	63 (55.3%)	41 (36.0%)		8 (7.0%)		2 (1.8%)				

## Discussion

This study addresses the paucity of research on the oral health profile of sugar mill workers, which reported a high prevalence of low OHL, inadequate attitudes, and poor oral health practices, all of which showed significant associations with adverse oral health outcomes, particularly dental caries and periodontal disease.

The proportion of participants with low OHL in this study was in line with the findings of Mailhe et al. [22] but contrasts with Khajuria et al. [23], where the majority of the participants had high OHL. This variation across studies could be attributed to differences in population demographics, educational background, and SES, underscoring the need to address OHL through tailored health promotion strategies. Although various tools exist to measure OHL, the HeLD-14 scale was preferred for its concise structure, multidimensional framework, and ease of administration, allowing more comprehensive evaluation of participants' OHL across multiple domains in a community setting [19].

In the present study, participants with limited OHL had high mean DMFT and CPI/LOA scores (p < 0.05), which coincides with the results of Khajuria et al. [23] and contrasts with the findings of Baskaradoss [5], who stated no significant differences between the participants with low and high OHL levels (p = 0.561). Similarly, the findings of the present study showed that more than half of the participants presented with an inadequate attitude toward oral health, which accords with the results of Reddy et al. [24], where the participants with a negative attitude reported gingival bleeding, calculus, and deeper periodontal pockets. This suggests that in addition to general health determinants, OHL serves as an intermediary factor in influencing oral health outcomes, health-related behaviors, and the utilization of dental services [2].

Pertaining to oral health behaviors, the current study findings showed that only 15% of the participants had the habit of brushing twice daily, which was in accordance with the study conducted by Pavithra et al. [10], who reported that those with improper oral hygiene practices had poor oral health outcomes. Of 260 participants, only 14.6% used additional oral hygiene aids, of which the tongue cleaning aid was most commonly used. None of them reported using other oral hygiene aids, such as dental floss or mouth rinse, reflecting the lack of awareness as well as knowledge regarding the use of additional oral hygiene aids among the population [25].

Sharma et al. [26] reported that nearly half of the participants (46.1%) used a horizontal brushing technique, whereas in the present study, only a smaller proportion (29.2%) practiced a similar method of brushing, while 26.9% did not follow any fixed method of brushing. Similarly, the differences in toothbrush replacement frequency when compared to An et.al. [27] follow a similar pattern, where most of the participants used to change their brush once every two to three months or six months. The low prevalence of proper brushing techniques suggests a lack of oral health awareness, underscoring the necessity of organized oral health education initiatives to reinforce proper oral hygiene practices.

With respect to dental service utilization, the majority of the participants had never been to a dentist within the past one year, which was inconsistent with the findings of Elkerdany et al. [28] and Henderson et al. [29], where the individuals with high OHL were more likely to have seen a dentist within the previous year. Therefore, strengthening the OHL and accessibility could help improve dental service utilization among the study population.

This study uniquely focuses on sugar mill workers, a population with distinct occupational exposures and potential oral health risks. Inclusion of sociodemographic, occupational, and behavioral factors enabled a comprehensive assessment of the determinants influencing OHL and outcomes. Assessing their attitudes and practices can help identify existing knowledge gaps and inform strategies to promote healthier oral health behaviors. However, the cross-sectional design of the study limits causal inference, and the reliance on self-reported data may introduce recall bias. Although occupational risk factors were addressed, the study did not quantify exposure levels, such as the concentration of sugar dust or duration of exposure, and their significance in oral health.

Recommendations

Future longitudinal studies are warranted to establish causal relationships and to further elucidate the long-term impact of OHL, attitude, and practices on oral health outcomes. In addition, the establishment of dental clinics within factory premises could substantially improve the oral health status of industrial workers by providing timely access to preventive, promotive, and curative services, thereby integrating oral health care into the broader occupational health system.

## Conclusions

This study concludes that adequate OHL was significantly associated with lower caries experience and better periodontal status among sugar mill workers. Similarly, favorable oral health attitudes and practices showed a positive influence on periodontal health. These findings underscore the need for strengthening workplace-based oral health promotion initiatives and suggest that policy-making and advocacy efforts may benefit from focusing on incorporating oral health into existing occupational health frameworks, thereby facilitating comprehensive health monitoring for industrial workers.

## Appendices

Supplemental material 1

**Table 7:** Oral health literacy questionnaire Health Literacy in Dentistry Scale (HeLD)-14 Scale [18].

S. no.	Questions	Unable (0)	Very difficult to perform (1)	Neutral (2)	With a little difficulty (3)	Without any difficulty (4)
1.	Ability to pay attention to your dental or oral health?					
2.	Ability to make time for things that are good for your dental or oral health?					
3.	Ability to read written information, for example, leaflets given to you by your dentist?					
4.	Ability to read dental or oral health information brochures left in dental clinics and waiting rooms?					
5.	Ability to take family or a friend with you to a dental appointment?					
6.	Ability to ask someone to accompany with you for a dental appointment?					
7.	Ability to pay while consulting a dentist?					
8.	Ability to pay for medication to manage your dental or oral health?					
9.	Ability to get a dentist appointment?					
10.	Do you know what to do to get a dentist appointment?					
11.	Ability to look for a second opinion about your dental health from a dental health professional?					
12.	Ability to use information from a dentist to make decisions about your dental health?					
13.	Ability to carry out instructions that a dentist gives you?					
14.	Ability to use advice from a dentist to make decisions about your dental health?					

Supplemental material 2

**Table 8:** Oral health-related attitude questionnaire

S. no.	Questions	Yes	No	Maybe
1.	Do you think that keeping your teeth clean and healthy is beneficial to your health?			
2.	Do you think that tooth decay require treatment?			
3.	Do you think that improper brushing leads to gum disease?			
4.	Do you think tobacco or betel nut chewing benefits your oral health?			
5.	Do you think that educating parents is important in preventing caries in children?			
